# Gut bacterial lauroyl tryptamine reduces GPR183-associated inflammatory responses in human macrophages and T cells *in vitro*

**DOI:** 10.3389/fimmu.2026.1878373

**Published:** 2026-10-15

**Authors:** Marco Lezzerini, Vera Spek, Ivan C.N. Fung, Michelle Jessy Müller, Aleksej Kubelka, Simon Sieber, Wouter J. de Jonge, Andrew Y.F. Li Yim, Anje A. te Velde, Jianbo Zhang

**Affiliations:** 1 Swammerdam Institute for Life Sciences, University of Amsterdam, Amsterdam, Netherlands; 2 Amsterdam Gastroenterology Endocrinology Metabolism (AGEM), Tytgat Institute for Liver and Intestinal Research, Amsterdam University Medical Centers, Academic Medical Center (AMC), Amsterdam, Netherlands; 3 Department of Chemistry, University of Zurich, Zurich, Switzerland

**Keywords:** bacterial metabolites, fatty acid amide, G protein coupled receptors, inflammatory bowel diseases, host-microbe interaction, mucosal immunity

## Abstract

**Introduction:**

Recent research has highlighted the role of gut microbiota in producing bioactive metabolites that interact with G-protein coupled receptors (GPRs) and influence immune responses. Lauroyl tryptamine (LT), a metabolite produced by *Agathobacter rectalis* (formerly *Eubacterium rectale*), has been reported to antagonize GPR183, a receptor implicated in inflammatory bowel diseases (IBD). LT inhibits the binding of GPR183 with its natural ligands 7a,25-dihydroxycholesterol (OHC).

**Methods:**

Here, we analyzed the *GPR183* gene expression in intestinal tissues from IBD patients and non-IBD controls, as well as our previous single-cell transcriptomic dataset to identify key GPR183-expressing cells. We then use in vitro THP-1 derived macrophage and Jurkat T cell culture to test the effects of LT on inflammatory responses.

**Results and discussion:**

The GPR183 gene expression is elevated specifically in colonic and rectal tissues of ulcerative colitis patients, but not in Crohn’s disease or ileal tissue. By analyzing single-cell transcriptomic dataset, we identified monocytes/macrophages, T cells, B cells, and innate lymphoid cells as key *GPR183*-expressing populations in human intestine. Using cell culture models, we demonstrated that LT modulates inflammatory pathways. In LPS-stimulated macrophages, OHC increased transcription of pro-inflammatory genes *IL6, TNFA*, and *IL1B*, which are reduced by LT. In activated Jurkat T cells, LT slightly decreased the OHC-mediated increase in the transcription of IL2. These findings support the hypothesis that LT, a known GPR183 antagonist, could modulate GPR183-associated inflammation in human macrophages and T cells in a context- and cell type-dependent manner, offering potential mechanistic insights into bioactive metabolite-mediated host-microbiota interactions in human intestine.

## Introduction

Alternations in the gut microbiota are strongly associated with inflammatory bowel diseases (IBD) (1–3). The relative abundance of several bacteria taxa, including *Agathobacter rectalis* (formerly *Eubacterium rectale*) and *Faecalibacterium duncaniae* (formerly *F. prausnitzii*), are lower in patients with IBD compared to healthy individuals (2, 3). Mechanistically, these bacteria may contribute to gut homeostasis through the production of bioactive metabolites including short chain fatty acids (4), secondary bile acids (5, 6), and tryptophan metabolites (7). For example, propionate and butyrate are agonist of G-protein coupled receptor 41 (GPR41) and GPR43 (8), and exhibit protective inflammatory responses in mice models of colitis and infection (4, 9), as well as in human cells (10, 11). Recently, Chang et al. (2021) identified fatty acid amide as a novel class of bioactive metabolites interacting with GPRs, of which lauroyl tryptamine, produced by *A. rectalis*, was reported to inhibit pro-inflammatory receptor GPR183 (also known as EBI2) with an IC50 of 0.98 μM (12).

GPR183 is a cell surface membrane receptor that constitutively signals through Gi (but not Gαs or Gαq) (13) and senses extracellular endogenous oxysterols. Among these oxysterols, 7α,25-dihydroxycholesterol (OHC) is the most potent known GPR183 ligand that can induce the proliferation, activation, and migration of both innate and adaptive immune cells (14). This OHC-GPR183 axis has been implicated in intestinal inflammation by promoting the migration of group three innate lymphoid cells (15) and decreasing IL-10 expression in regulatory T cells (16). GPR183 has also been linked with IBD (17). Genome-wide association studies have hinted *GPR183* as a potential risk gene for IBD, as the nucleotide polymorphism (rs9557195) in *GPR183* leads to increased expression of the receptor in lymphocytes (18). In addition, *GPR183* is required for ILC3-mediated protection against *Citrobacter rodentium* by influencing the localization of ILC3s in the lamina propria (19). In B cells, *GPR183* directs cell migration and positioning within the outer follicle (20). Consequently, *GPR183* expression by activated B cells promotes their migration to extrafollicular sites and supports early plasmablast responses (21). In addition to adaptive immunity, the GPR183-oxysterol axis promotes the development of intestinal lymphoid structures and colitis (22).

Here, we investigated the expression of *GPR183* in the intestinal tissue of IBD and non-IBD individuals at both tissue and single cell level. We identified monocytes, macrophages, and CD4^+^ T cells with intermediate-to-high *GPR183* expression. We then examined whether the gut bacteria-derived LT modulate OHC-GPR183-associated inflammatory responses in THP-1-derived macrophages and Jurkat T cells, and revealed that LT reduces the transcription of *IL6, TNFA*, and *IL1B* but not NF-κB pathway in LPS-stimulated macrophages, while decreases the transcription of *TNFA, IL1B*, and *IL2* in activated T cells.

## Materials and methods

### Synthesis of lauroyl tryptamine (LT, N-(2-(1H-indol-3-yl)ethyl)dodecanamide)

Unless otherwise stated, all chemicals were of reagent grade and purchased from Sigma-Aldrich-Merck. Reactions were monitored for completion by analyzing a small sample (after suitable workup) by thin-layer chromatography (TLC) or UHPLC-MS. Solvents for reactions were of p.a. grade or distilled prior to their use. Extracts were dried over anhydrous MgSO_4_ or NaSO_4_. Evaporation of the solvents *in vacuo* was done with the rotary evaporator at a bath temperature of 40 °C unless stated otherwise. TLC: *Merck* TLC plates silica gel *60* on glass with the indicated solvent system; the spots were visualized by UV light (254 nm) and exposure to stains of KMnO_4_. IR spectra: *Varian 800 FT-IR ATR Spectrometer*; applied as neat samples; 1/*λ* in cm^−1^. ^1^H-NMR spectra: *Bruker AV2-501* (500 MHz); *δ* in ppm rel. to solvent residual peak), *J* in Hz. ^13^C-NMR spectra: *Bruker AV2-501* (125 MHz); *δ* in ppm rel. to solvent residual peak). High-resolution electrospray mass spectra (HR-ESI-MS) were recorded on a *maXis* QTOF-MS instrument (*Bruker Daltonics* GmbH, Bremen, Germany). The samples were dissolved in CH_3_OH at a concentration of ca. 50 µg/ml and analyzed via continuous flow injection (2 µL/min). The mass spectrometer was operated in the negative electrospray ionization mode at -4,000 V capillary voltage, 500 V endplate offset, with a N_2_ nebulizer pressure of 0.8 bar and dry gas flow of 4 l min^-1^ at 180 °C. Mass spectra were acquired in the mass range from *m/z* 50 to 2,000 at 20,000 resolution (full width at half maximum) and 1.0 Hz rate. The mass analyzer was calibrated between *m/z* 118 and 2,721 using an *Agilent* ESI-L low concentration tuning mix solution (*Agilent*, USA) at a resolution of 20,000 and a mass accuracy below 2 ppm. NMR spectra and HR-MS suggest the structure of synthetized LT is identical to the bacterial metabolite (12).

LT was synthesized following a modified reported procedure (23). Lauric acid (Sigma-Aldrich 8.05333, 3.12 mmol, 625 mg, 1.0 equiv.), tryptamine (Sigma-Aldrich 193747, 3.12 mmol, 500 mg, 1.0 equiv.), EDCI·HCl (Sigma-Aldrich E6383, 3.74 mmol, 718 mg, 1.2 equiv.), HOBT (Sigma-Aldrich 157260, 3.75 mmol, 506 mg, 1.2 equiv.) were dissolved in dichloromethane (Sigma-Aldrich D65100, 15 mL) at 0 °C, then Et_3_N (Sigma-Aldrich T0886, 1.52 mL, 3.5 equiv.) was added. The reaction mixture was allowed to warm up to room temperature and was stirred for 18 h. After full conversion of the reaction, based on UHPLC-MS, the solvent was removed by rotary evaporation under reduced pressure. The resulting crude was purified by silica gel flash column chromatography (DCM: MeOH, 95:5) to afford the desired product as a brown solid. The product was recrystallized (MeOH/Hexane, 5:100), followed by purification by preparative RP-HPLC (Gemini NX, C18, 5 μm, 110 Å, 250 mm × 21.2 mm, solvent A: H_2_O + 0.1% HCOOH, solvent B: CH_3_CN + 0.1% HCOOH, 20 mL/min; LC time program (time –% B): 5 min – 5%, 30 min – 95%, 30.5 min – 100%). The combined fractions were freeze-dried to obtain lauroyl tryptamine (634 mg,1.85 mmol, 59%, t_R_ = 15.2 min) as a white solid. The characterization data are in agreement with those reported. R*_f_* = 0.3 (CH_2_Cl_2_: CH_3_OH, 95:5). FTIR: $\tilde{\nu }=3391$, 3261, 3086, 2954, 2918, 2872, 2850, 1650, 1629, 1558, 1468, 1456, 1425, 1377, 1359, 1339, 1299, 1281, 1253, 1224, 1210, 1126, 1094, 1069, 1011, 930, 912, 880, 847, 801, 765, 739, 721, 672, 608, 580, 560, 495 cm^-1^. ^1^H NMR (400 MHz, CDCl_3_) *δ* 8.04 (s, 1H), 7.62 (m, 1H), 7.38 (dt, *J* = 8.2, 1.0 Hz, 1H), 7.22 (ddd, *J* = 8.2, 7.1, 1.2 Hz, 1H), 7.13 (ddd, *J* = 8.0, 7.0, 1.1 Hz, 1H), 7.04 (m, 1H), 5.47 (s, 1H), 3.61 (td, *J* = 6.7, 5.8 Hz, 2H), 2.98 (td, *J* = 6.7, 0.9 Hz, 2H), 2.09 (t, *J* = 7.7 Hz, 2H), 1.64 – 1.52 (m, 2H), 1.34 – 1.22 (m, 16H), 0.87 (t, *J* = 6.8 Hz, 3H). ^13^C NMR (101 MHz, CDCl_3_) *δ* 173.3, 136.6, 127.5, 122.3, 122.2, 119.6, 118.8, 113.1, 111.4, 39.8, 37.0, 32.0, 29.7, 29.7, 29.6, 29.5, 29.5, 29.4, 25.9, 25.5, 22.8, 14.2. ESI-HRMS (MeOH): *m/z* 341.25988 (C_22_H_33_N_2_O^–^; [M-H]^–^, calc. 341.25984).

### Cell culture and maintenance

The THP-1 human monocyte leukemia cell line (ATCC TIB-202) and Jurkat T cells were cultured at a density of 3×10^5^ cells/mL in complete growth medium consisting of RPMI-1640 medium with L-glutamine (1187505, Gibco), 1% penicillin-streptomycin (P4333, Sigma-Aldrich), 1% GlutaMAX (35050061, Gibco), and 10% heat-inactivated fetal bovine serum (F9665, Sigma-Aldrich), in a Forma Series 3 Water Jacket CO_2_ Incubator (Thermo Fisher) at 37 °C and 5% CO_2_. The cells were passaged every two to three days by centrifugation, followed by resuspension in a microbiological safety cabinet (51026638, Thermo Fisher). Before passaging, the seeding density was calculated by counting the cells with the Countess II (Invitrogen AMQAX1000). For this automated counting method, an equal volume of the cell suspension was mixed with a 0.4% trypan blue solution (T10282, Invitrogen). Then, 10 µL of the mixture was pipetted into a Countess chamber slide (C10228, Invitrogen). Cells were visually checked regularly under a microscope (DMI3000B, Leica) by taking either the entire 25 cm² cell flask (690160, Greiner Bio-One) or 100 µL into a 96-well plate (391-3325, Greiner Bio-One).

### Macrophage differentiation and treatment

THP-1 cells were seeded at 5 ×10^5^ per ml per well in 24-well plates (Greiner bio-one, 662102) and differentiated into M0 macrophages by treatment with 100 nM PMA (500582, Sigma-Aldrich) for three days. The macrophages were then washed with DPBS (10010023, Gibco) and treated with 0.23 nM OHC (700080P, Avanti), 9.8 µM lauroyl tryptamine, both (0.23 nM OHC and 9.8 µM lauroyl tryptamine), DMSO (vehicle control), or positive control (1 µM GSK682753A, MedChemExpress, 1334294-76-6). 100 ng/mL LPS (L4516, Sigma-Aldrich) was added simultaneously to stimulate the macrophages. Cells were collected after 24 h of incubation at 37 °C and 5% CO2 for RT-qPCR analysis.

### Jurkat T cell treatment

Jurkat cells were activated with 10 ng/ml of PMA for 6 h and subsequently exposed to 0.23 nM OHC, 9.8 µM lauroyl tryptamine, both compounds (0.23 nM OHC and 9.8 µM lauroyl tryptamine), DMSO vehicle control. Cells were collected after 24 h of incubation at 37 °C and 5% CO2 for RT-qPCR analysis.

### Preparation of cDNA

Approximately 4 µg of RNA was isolated from 5 × 10^5^ cells, using the RNeasy Mini Kit (74104, Qiagen) according to the manufacturer’s protocol. Here, 350 μL of buffer RLT was used to disrupt the cells and on-column DNase digestion was performed after the first washing step. For the DNase digestion step, 80 µL of the DNase I incubation mix was added to the RNeasy spin column. The column was then incubated for 15 minutes at room temperature. Then, 350 μL of RW1 buffer was added, the column was spun down at 8000 x g, and the flow-through was discarded. The residual genomic DNA was removed using DNA-free™ DNA Removal Kit (AM1906, Invitrogen). Briefly, 40 μL of the RNA sample was mixed with 4 μL of DNase I buffer and 1 μL of rDNase I, and the mixture was incubated for 25 minutes at 37 °C. Then, 4 μL DNase Inactivation Reagent was added and incubated for 2 minutes at room temperature. The samples were spun down at 10,000 g and supernatant was collected. The RNA concentration was determined using a DS-11+ spectrophotometer (Denovix). Here, the spectrophotometer was calibrated using 2 μL of MQ water, after which RNA concentration was determined by pipetting 2 μL on the machine. Afterwards, RNA samples were stored at –80 °C. For cDNA synthesis, 500 ng RNA was reverse-transcribed using the High-Capacity cDNA Reverse Transcription Kit (4368814, Applied Biosystems), according to the manufacturer’s instructions. The thermocycler (TProfessional Thermocycler, Biometra) was programmed as follows: 10 minutes at 25 °C, 120 minutes at 37 °C and 5 minutes at 85 °C, ending at 4 °C. Negative controls without reverse transcriptase were included. The cDNA was then stored at –20 °C.

### Reverse transcription quantitative polymerase chain reaction (RT-qPCR)

For the RT-qPCR, 10 or 2.5 ng of cDNA was used with 10 μL of SYBR™ Green qPCR Master Mix (A25742, Applied Biosystems) with a total reaction volume of 20 µL and two technical replicates. Primers were added in a concentration of 500 nM, with *ACTB* or *GAPDH* as reference genes. The primers used are listed in **Supplementary Table S1**. The Real-Time PCR machine (Applied Biosystems 7300 Real-Time PCR System) was programmed as follows: 2 minutes at 50 °C, 10 minutes at 95 °C, 40 cycles of 15 seconds at 95 °C and 1 minute at 60 °C, followed by a disassociation step of 15 seconds at 95 °C, 1 minute at 60 °C, 15 seconds at 95 °C and 15 seconds at 60 °C. The relative gene expression was calculated using the 2^-ΔΔCt^ method, with expression normalized to the indicated reference gene and subsequently expressed relative to the vehicle-control condition. Technical duplicate Cq values were averaged before calculation and statistical analysis.

### Single cell transcriptomic analysis

Single-cell RNA-sequencing data from 9 UC and 5 non-UC patients were obtained from Fung et al. (24) In short, colonic biopsy samples were obtained from patients during ileocolonoscopy at the Department of Gastroenterology and Hepatology of the Amsterdam UMC. All patients were ≥16 years old. Samples were imported, processed, and analyzed in the R programming environment (v4.2.1) using Seurat (v4.3.0). Gene expression was normalized by SCTransform using default parameters. Cell annotations were used from the original manuscript as-is, which were annotated in a semi-automatic fashion using multimodal reference-mapping against a peripheral blood mononuclear cell (PBMC) cellular indexing of transcriptomes and epitopes sequencing (CITE-seq) (25), and a subsequent manual curation using canonical markers.

### Statistics

One-way ANOVA analysis with Tukey’s multiple comparisons test was performed for qPCR data to compare the differences between the treatment groups.

## Results

### Expression of *GPR183* in intestinal tissues of IBD is disease- and site-specific.

To understand the expression pattern of GPR183 gene in the IBD intestinal tissue, we leveraged the IBD Transcriptome and Metatranscriptome Meta-Analysis (IBD TaMMA, https://ibd-meta-analysis.herokuapp.com/). Analysis of GPR183 mRNA expression in mucosal intestinal tissue from IBD patients and non-IBD controls revealed a distinct, site-specific pattern. The expression of *GPR183* in colonic and rectal tissues of UC patients was significantly higher than the non-UC control, while no difference, as expected, was observed in the ileum in UC vs control (**Figure 1A**). This pronounced increase suggests a specific role for *GPR183* in UC. In contrast, *GPR183* expression showed no significant changes for CD. The expression of *GPR183* in ileum, colon, and rectum of CD patients was not significantly different to non-IBD controls (**Figure 1B**). These results highlight a marked and statistically robust upregulation of *GPR183* specifically in UC, contrasting with its unaltered expression in CD across ileal, colonic, and rectal tissues.

**Figure 1:**
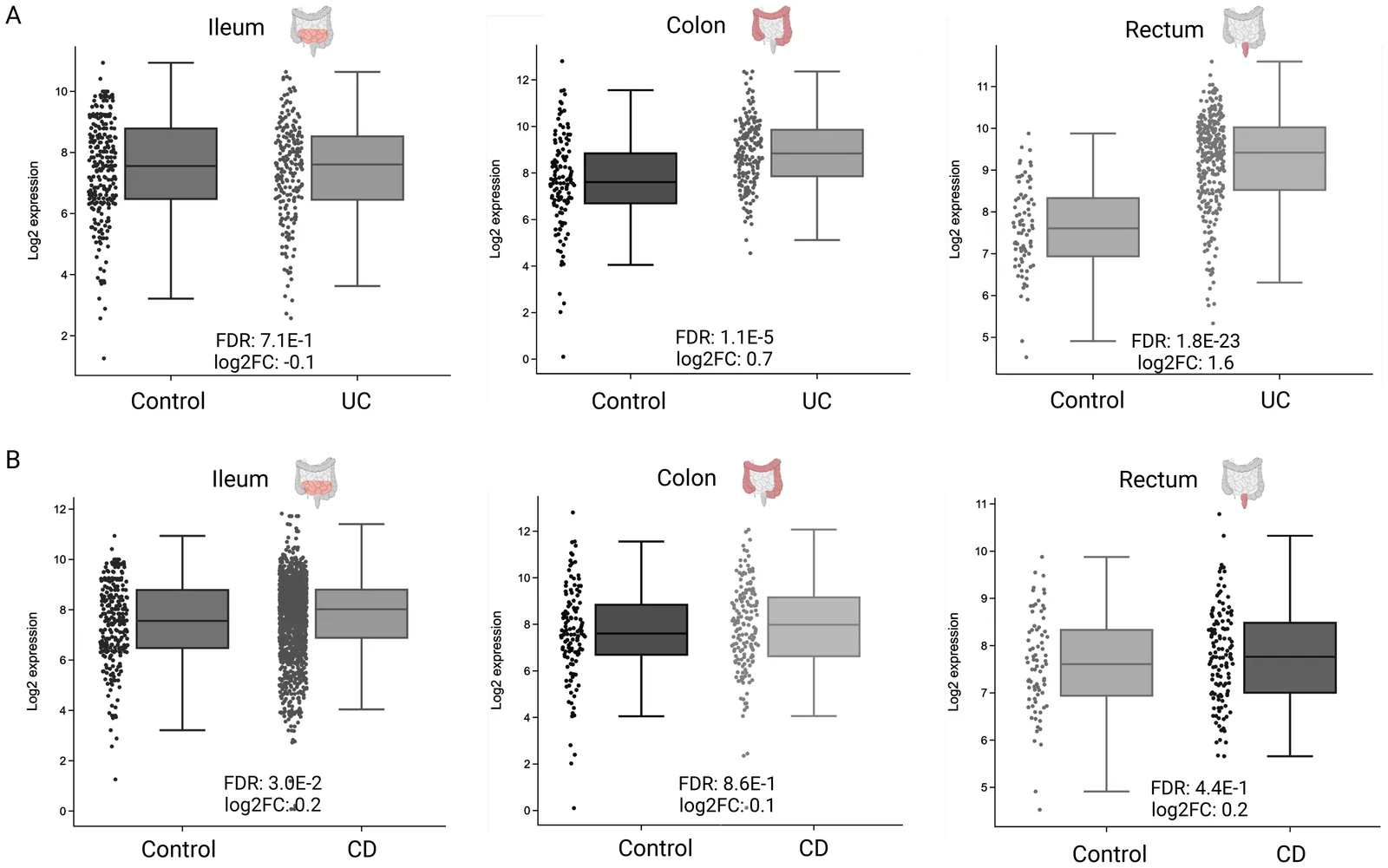
Tissue- and disease-specific higher expression of gene *GPR183* in inflammatory bowel diseases ulcerative colitis (UC) and Crohn’s disease (CD). **(A)** expression of *GPR183* in ileal, colonic, and rectal tissue of UC patients versus control. **(B)** expression of *GPR183* in ileal, colonic, and rectal tissue of CD patients versus that from non-IBD controls. Datasets are from IBD TaMMA https://ibd-meta-analysis.herokuapp.com, a web app that compiles transcriptomic datasets from IBD patients and non-IBD individuals. Each dot represents one tissue sample from indicated gastrointestinal tract location.

### Cell-type specific expression of *GPR183* in intestinal immune cells.

To gain a high-resolution expression pattern of *GPR183* across different types of immune cells in the human intestine, we interrogated a single cell transcriptome dataset of intestinal tissue from patients with UC (**Figure 2A**) (24).Among the *PTPRC*+ immune cells, we observed the highest levels of *GPR183* expression amongst CD4^+^ TEM, Treg, CD4^+^ TCM, and ILC, followed by MAIT cells, B cells, naïve CD4^+^ cells, and monocytes (**Figure 2B**). Interestingly, among monocytes in a different dataset (26, 27)., the high expression of *GPR183* was only observed in classical monocytes but not in non-classical monocytes (**Figure 2C**). B cells showed high expression, which is in agreement with previous studies that GPR183 protein is essential for the migration of B cells (28, 29) and formation of follicles in the intestine (15, 22). Monocytes, ILCs, and T cells, in particular CD4^+^ T cells, showed similar levels of *GPR183* expression. Taken together, *GPR183* is highly expressed among intestinal immune cell populations and is cell-type-dependent, suggesting that these *GPR183*-expressing immune cells may respond to GPR183 antagonists produced by gut bacteria.

**Figure 2:**
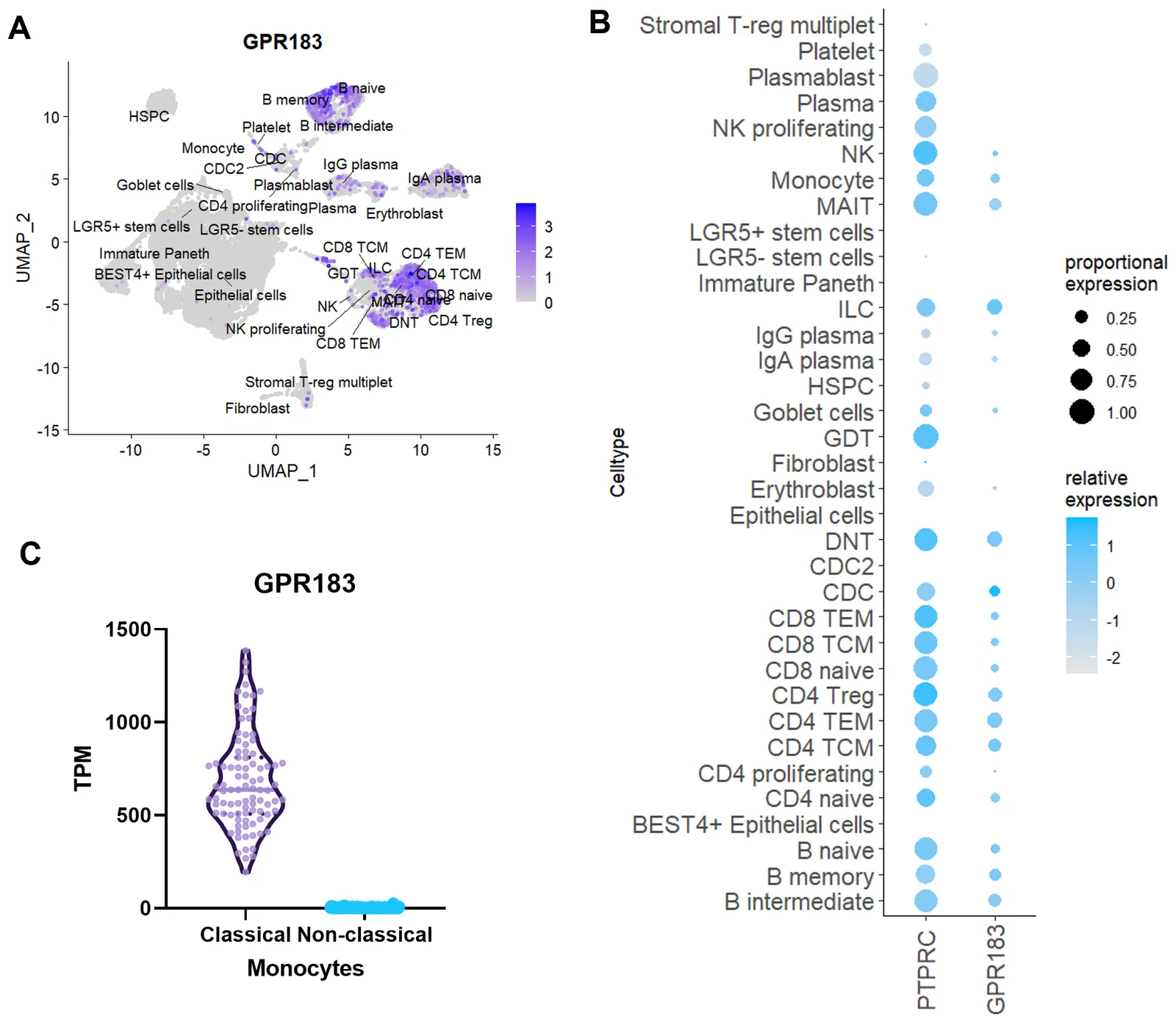
Presence of *GPR183* in human intestinal mucosa from UC and non-UC patients. **(A)** Uniform manifold approximation projection (UMAP) visualization of cell types and the expression pattern of *GPR183* visualized in intestinal biopsies obtained from 9 UC patients and 5 non-UC patients. **(B)** Relative mRNA expression of *GPR183* in different types of cells in human colonic mucosa. Proportional expression: percentage of cells that have positive expression of indicated gene. Relative expression: the expression level in IBD patients relative to non-IBD control samples. **(C)** mRNA levels of *GPR183* in classical and non-classical monocytes. Data were obtained from previously reported datasets (26, 27). TPM: transcript per million.

### Gut bacterial GPR183-antagonist lauroyl tryptamine (LT) inhibits inflammation in THP-1 monocytes and macrophages

Gut microbes such as *E. rectale* were found to produce lauroyl tryptamine (LT). LT is a competitive antagonist of OHC at *GPR183*. Reasoning that monocytes highly express *GPR183* (**Figure 2**), we sought to evaluate the effects of LT on the transcription of the *GPR183* and inflammatory responses in monocyte-differentiated macrophages. In non-stimulated macrophages, LT did not affect the transcription of *GPR183*. To test if LT affects the inflammation state of non-stimulated macrophages, we examined the anti-inflammatory gene *IL10* and several pro-inflammatory genes, including the NF-κB pathway *NFKBIA* and *RELA*, as well as TNF pathway genes *TNFA*, *CXCL8*, *IL1B*, and *IL6*. The transcription of *RELA* and *NFKBIA* did not differ significantly among treatments including OHC, LT, and OHC+LT. Interestingly, OHC significantly increased the transcription of *IL10* (p<0.05, One-way ANOVA; **Supplementary Figure S2**), which is abolished by LT treatment, indicating potential interaction between OHC and LT via GPR183. Moreover, LT alone increased the expression of pro-inflammatory genes *CXCL8, IL1B*, and *TNFA*, while LT-OHC cotreatment did not change this trend. These results suggest that OHC did not significantly alter expression of the measured inflammatory genes under unstimulated conditions, and little antagonism by LT occurred.

Next, we sought to determine the impact of LT on OHC-GPR183 axis in activated immune cells. In THP-1 cells, 24-h LPS stimulation increased the transcription of proinflammatory genes *IL1B, IL6*, and *TNFA* by 3–60 fold in THP-1 monocytes, while slightly increased the expression of *GPR183* (**Supplementary Figure S3**), suggesting that cells are successfully activated upon LPS treatment. LPS induces the secretion of IL-1ß, which is not changed by OHC treatment, but attenuated by LT alone or OHC+LT co-treatment (**Supplementary Figure S3**). To investigate the effects of LT on the transcription of pro-inflammatory cytokine genes in LPS-activated immune cells, we differentiated monocytes into M0 macrophages, after which cells were stimulated with LPS and simultaneously exposed to different compounds (**Figure 3A**): i.e., DMSO (vehicle control), OHC, LT, OHC+LT, and OHC+GSK [positive control, GSK682753A, a known GPR183 inverse agonist (12)]. In LPS-activated macrophages, the transcription of *GPR183* was slightly elevated in the presence of LT, OHC+LT, or OHC+GSK (**Figure 3B**). For the anti-inflammatory cytokine gene *IL10*, no significant difference was observed between the treatments, with high variation among the samples. This is likely due to the low expression of *IL10* under LPS stimulation, with Cq value of *IL10* around or above 35 (data not shown), which is considered the limit of reliable quantification. Similarly, the expression of pro-inflammatory genes *RELA* and *NFKBIA* did not change significantly upon different treatments including the known GPR183 inverse agonist GSK682753A (**Supplementary Figure S4**), indicating that GPR183 antagonism has marginal effects on NFKB pathway upon activation of OHC. In contrast, for the other pro-inflammatory genes, LT and GSK antagonism showed different levels of anti-inflammatory effects. For instance, *CXCL8* expression was increased upon LT treatment, but significantly decreased in the presence of LT and OHC. For *IL1B* and *TNFA*, LT-OHC cotreatment was more than 35% lower than with OHC alone, although the difference was not statistically significant (**Figure 3C**). Finally, OHC did not further increase the transcription of *IL6* upon LPS stimulation, while LT treatment alone, OHC-LT co-treatment, and OHC-GSK co-treatment, all were associated with lower *IL6* expression (**Figure 3C**). Together, these results support that LPS significantly increases the inflammation state of the macrophages, and this inflammation can be maintained by GPR183 agonist OHC but reduced by microbiome-derived GPR183 antagonist LT.

**Figure 3:**
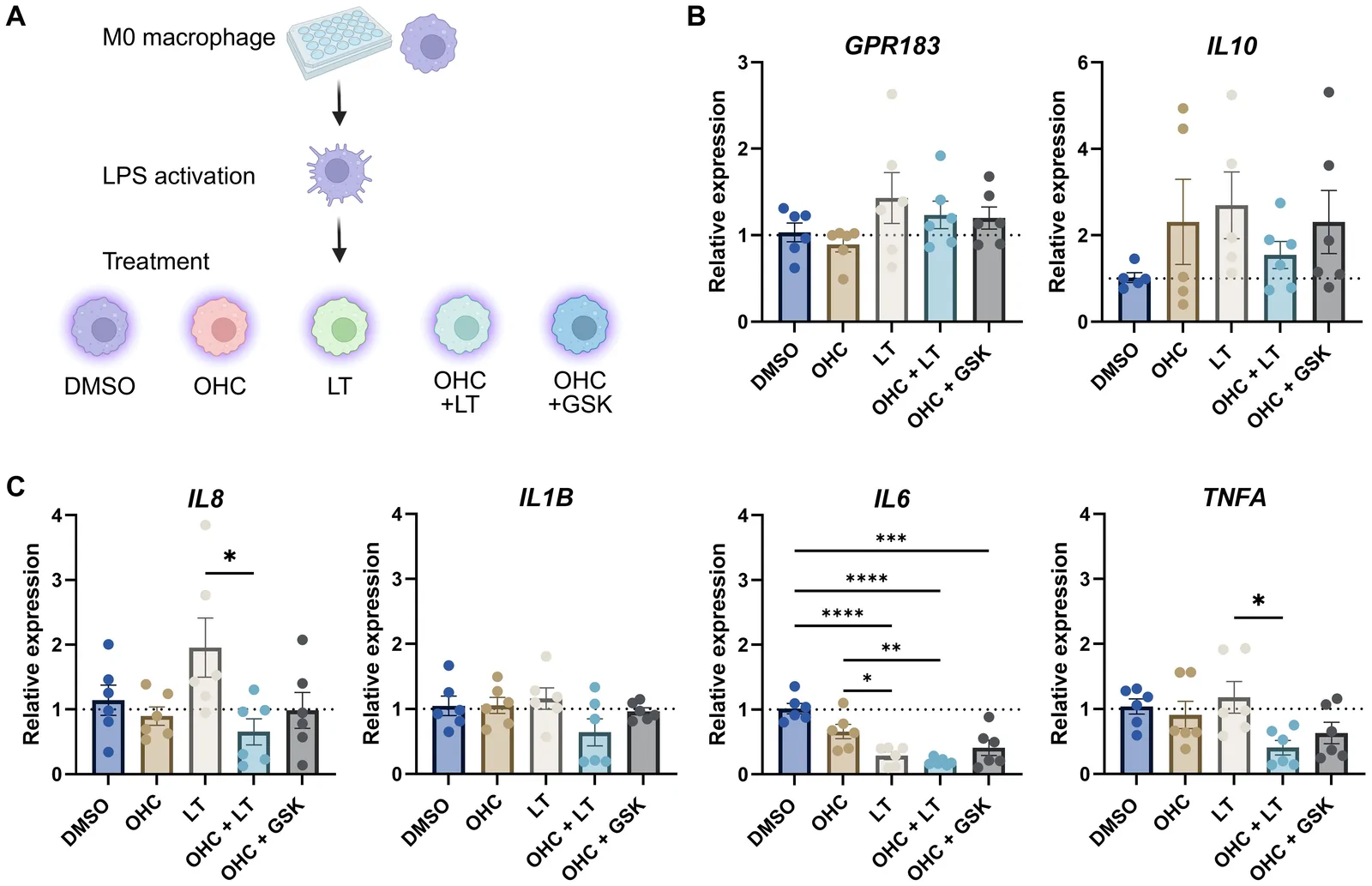
Effects of lauroyl tryptamine in LPS-stimulated M0 macrophages. **(A)** Experimental workflow of macrophage differentiation, activation, and treatment. **(B)** Expression of genes *GPR183* and *IL10* after 24-h treatment of LT, OHC, OHC+LT, or OHC+GSK. Values are relative to DMSO as the vehicle control, while GSK682753A, a known potent GPR183-specific antagonist [IC50 between 2.6 - 53.5 nM (30)], serves as a positive control. **(C)** Expression of genes *TNFA*, *CXCL8*, *IL1B*, and *IL6* in macrophages after the same treatments as **(A)**. Data represent six biological replicates per condition, obtained as three independently treated samples in each of two independent experiments. Error bars represent means and standard deviations. Technical qPCR duplicates were averaged before analysis. * P< 0.05, ** P< 0.01, *** P< 0.001, **** P< 0.0001 ordinary one-way ANOVA analysis with Tukey’s multiple comparisons test. OHC: 0.23 nM 7α, 25- dihydroxycholesterol. LT: 9.8 µM lauroyl tryptamine approximately 10-fold as the reported IC50 of 0.98 µM (12). GSK: 1 µM GSK682753A. All treatments contained the same amount of DMSO (0.16% dimethyl sulfoxide). During the treatment, 100 ng/mL LPS was added simultaneously for all conditions.

### LT impacts the *IL1B* and *IL2* in Jurkat T cells.

Next, we sought to interrogate the impact of LT on inflammation in T cells, as intestinal T cells are important for the mucosal immunity (31, 32) and express intermediate-to-high level of *GPR183* (**Figure 2**). Jurkat T cells were activated by PMA and then exposed to OHC, LT or their combination (**Figure 4A**). Upon activation by PMA, the transcription of all the tested genes in Jurkat T cells were substantially increased (**Figures 4B, C**). For example, *GPR183* was increased by about 100 fold, while other pro-inflammatory genes, i.e. *TNFA*, *IL2*, and *IL1B*, were increased by 50–160 fold, suggesting that cells were activated. Compared to PMA-activated cells, OHC treatment did not significantly change the expression of these genes, possibly due to the high activation states induced by PMA. In contrast, this proinflammatory activation is attenuated by LT treatment, as shown by reduced increase in the elevated expression of *IL1B*, *IL2*, and *GPR183* (**Figures 4B, C**). Interestingly, the negative regulator of inflammation NFKB inhibitor, i.e. *NFKBIA*, was slightly increased upon LT treatment (**Figure 4C**). These data suggest that LT does not completely abolish the pro-inflammatory activation of Jurkat T cells, albeit decreases the expression of *IL1B* and *IL2*. Previously, GPR183-mediated T cell migration and differentiation was augmented by dendritic cell-regulated IL2 at the interface of the B-cell follicle and T zone (33). Here, we showed that GPR183 antagonist LT has a direct impact on *IL2* expression in T cells.

**Figure 4:**
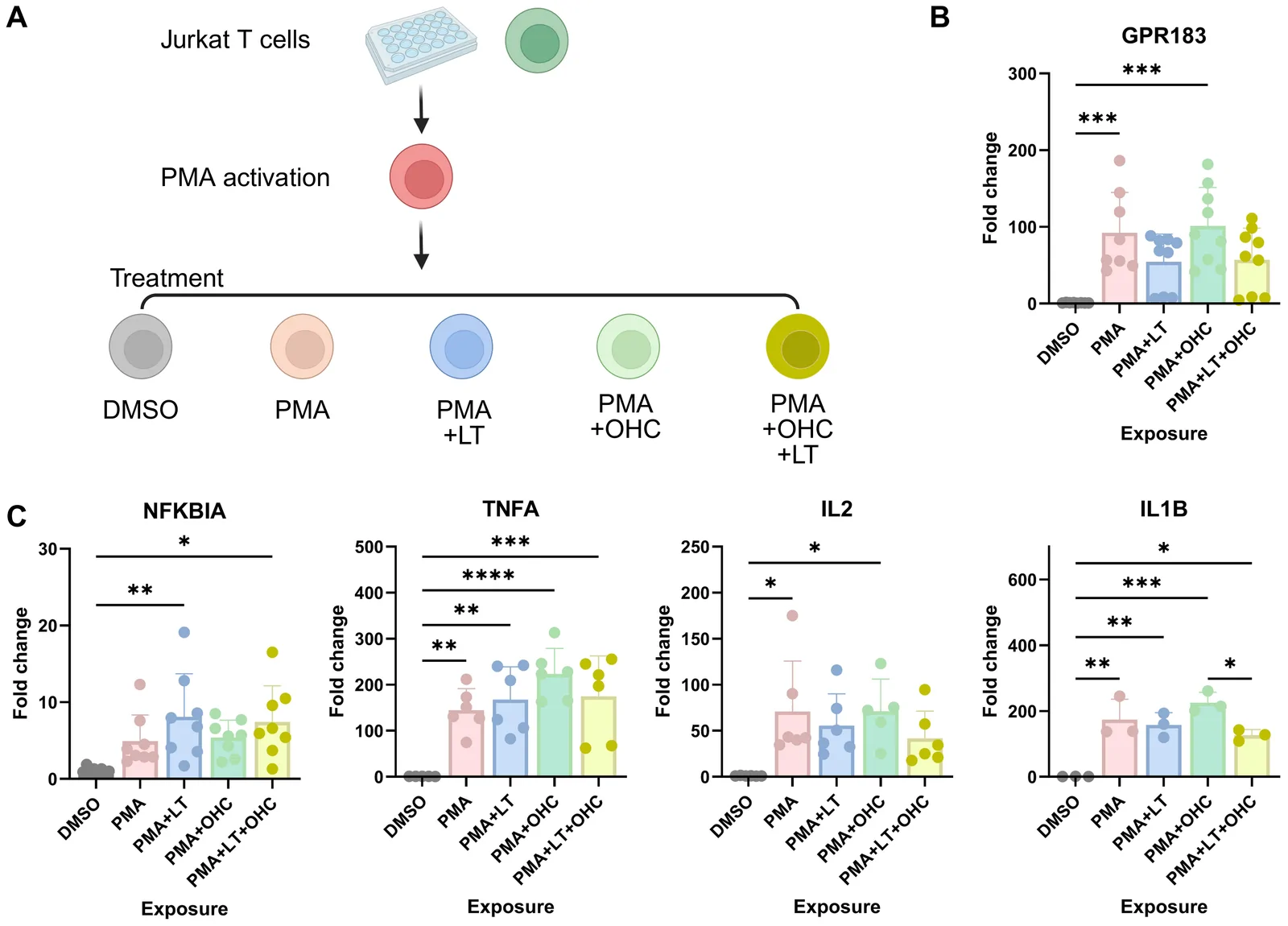
Influence of LT on Jurkat T cells. **(A)** Experimental workflow of T cell activation and treatments. **(B, C)** expression of *GPR183* **(B)**, and inflammation-related genes **(C)** after exposure of PMA, LT, OHC, and their combinations, relative to DMSO control. Data represent 3–9 biological replicates per condition, obtained as 1–3 independently treated samples in each of three independent experiments. Error bars represent means and standard deviations. Technical qPCR duplicates were averaged before analysis. * P< 0.05, ** P< 0.01, *** P< 0.001, **** P< 0.0001 ordinary one-way ANOVA analysis with Tukey’s multiple comparisons test.

## Discussion

We identified a disease- and site-specific upregulation of *GPR183* in human IBD, with pronounced elevation in colonic and rectal mucosa in ulcerative colitis but not in Crohn’s disease or ileal tissue. At single cell resolution, we mapped *GPR183* expression to intestinal monocytes/macrophages, CD4^+^ T cell subsets, and B cells, identifying the immune populations most likely to sense GPR183 ligands in the human gut. Building on the identification of lauroyl tryptamine as a bacterially produced GPR183 antagonist (12), we showed that this metabolite counteracts oxysterol-driven upregulation of pro-inflammatory cytokine genes in human macrophages and dampens *IL1B* transcriptional responses in activated T cells. This reduction of GPR183-associated inflammatory pathways by LT is aligned with the depletion of LT-producing bacterium *A. rectalis* (a.k.a. *E. rectale*) in IBD patients (3). These data support a model in which host-derived oxysterols and microbiota-derived fatty acid amides act as “accelerator” and “brake” of *GPR183*-mediated inflammatory signaling at the mucosal interface (**Figure 5**). While our results provide a mechanistic link between a gut bacterial metabolite LT and *GPR183*-associated inflammation in human intestinal immune cells, they are not yet validated in primary immune cells or using other advanced gut models (34). Future *in vivo* validation studies in UC and interrogation of other GPR183-expressing cell populations such as B cells and ILCs will be important to determine whether modulating oxysterol-GPR183-LT axis can be leveraged therapeutically to restore mucosal immune homeostasis.

**Figure 5:**
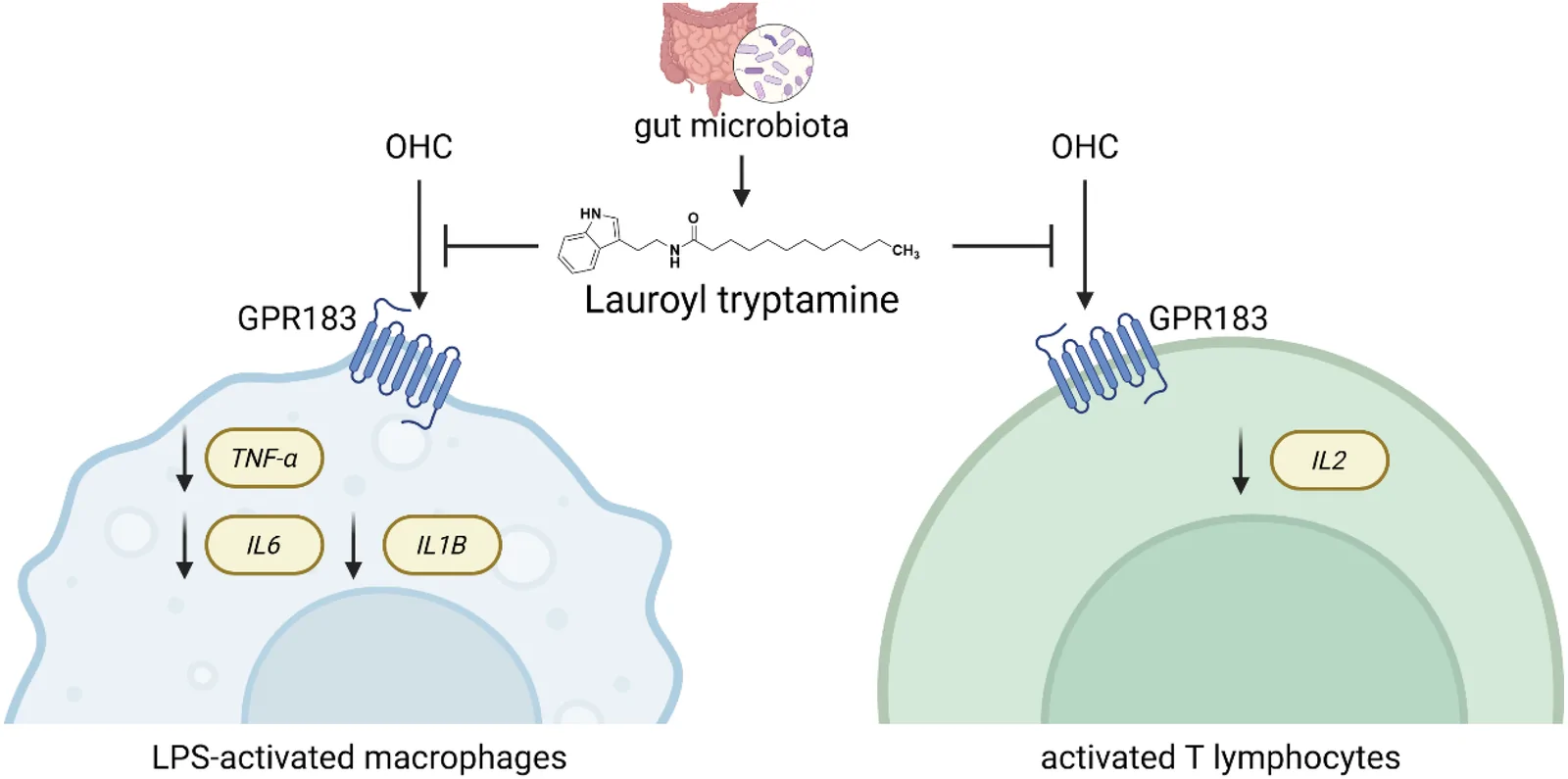
Model of LT’s effects on macrophages and T cells.

Bacterial metabolite LT can reduce the transcription of pro-inflammatory genes *TNFA*, *IL6*, and *IL1B* in activated macrophages, while decreasing *IL2* pathway gene expression in activated T cells. *TNFA* and *IL1B* regulate the expression of *IL6* at the transcriptional level. The concurrent decrease of *TNFA*, *IL1B* and *IL6* after LT treatment, together with the reported antagonism of GPR183 by LT (12), support the hypothesis that GPR183 antagonists can modulate *IL1B-TNFA-IL6* axis downstream of OHC-GPR183 pathway. However, we did not directly demonstrate the receptor dependence via GPR183 gene knockdown or knockout, and LT also increased the expression of *CXCL8* and *IL1B* in non-stimulated macrophages, indicating that its effects are context-dependent and may not be exclusively mediated by GPR183.

In Jurkat T cells, PMA activation markedly upregulated *IL1B*, *IL2* and *GPR183*, and LT had mild effects. So our data do not yet support a strong anti-inflammatory role of LT in T cells. Nonetheless, our single-cell analysis shows high GPR183 expression in intestinal CD4^+^ T cells, and prior studies have implicated GPR183 and oxysterols in Th17 differentiation (35), activation of CD4^+^ T cells (36) and positioning of activated CD4^+^ T cells at the interface of the follicle and T zone (15, 33). Taken together, these observations suggest that LT and related bacterial fatty acid amides are candidate modulators of GPR183-depdent cytokine pathways in human intestinal immune cells. Future work using primary cells and genetic perturbation of GPR183 will be required to establish this mechanism.

In summary, our analysis demonstrates a disease- and site-specific upregulation of GPR183 in human inflammatory bowel diseases, with pronounced increase in colonic and rectal mucosa from ulcerative colitis patients but not from Crohn’s disease patients or ileal tissue. At single cell resolution, we identified CD4^+^ T-cell subsets, monocytes/macrophages, B cells and ILCs as the main GPR183-expressing immune populations in the human intestine, defining the cell types most likely to sense GPR183 ligands from gut microbiome *in vivo*. In THP1-derived macrophages, the gut bacterial metabolite LT attenuates LPS or OHC-associated inflammatory gene expression stimulated macrophages, while increases pro-inflammatory gene expression in non-stimulated macrophages, exerting context-dependent effects. Together, these findings support a hypothesis in which host oxysterols and microbiota-derived GPR183 ligands can alter inflammatory programs in *GPR183*-high intestinal immune cells, and they provide a human tissue and cell-based framework for future mechanistic and *in vivo* validations.

## Data Availability

The datasets presented in this study can be found in online repositories. The names of the repository/repositories and accession number(s) can be found below: https://doi.org/10.5281/zenodo.14236827, zenodo.14236827.
