# Supplementary material for: Gut bacterial lauroyl tryptamine reduces GPR183-associated inflammatory responses in human macrophages and T cells in vitro
Source: Front Immunol. 2026 Oct 15;17:1878373. doi: 10.3389/fimmu.2026.1878373 (PMC13716806; doi:10.3389/fimmu.2026.1878373)
Supplement: Supplementary file 1 [file SupplementaryFile1.docx]

Supplementary Information

**Gut bacterial lauroyl tryptamine reduces GPR183-driven inflammation in human macrophages and T cells**

Marco Lezzerini,^1^ Vera Spek,^1^ Ivan I.C.N. Fung,^2^ Michelle Jessy Müller,^3^ Aleksej Kubelka,^2^ Simon Sieber,^3^ Wouter J. de Jonge,^2^ Andrew Li,^2^ Anje te Velde,^2^ Jianbo Zhang^1,2^

1 Swammerdam Institute for Life Sciences, University of Amsterdam, 1098XH Amsterdam, The Netherlands

2 Tytgat Institute for Liver and Intestinal Research, Amsterdam University Medical Centers, location Academic Medical Center (AMC). Meibergdreef 69-71, 1105BK Amsterdam, The Netherlands

3 Department of Chemistry, University of Zurich, 8057 Zurich, Switzerland

Correspondence: JZ j.zhang6@uva.nl

**Figure S1. NMR spectrum of lauroyl tryptamine.** (A) ^1^H-NMR Spectrum of lauroyl tryptamine in CDCl_3_ (400 MHz). (B) ^13^C-NMR Spectrum of lauroyl tryptamine in CDCl_3_ (125 MHz).

**Figure S2. Effects of LT on *GPR183* and pro-inflammatory cytokine genes in non-stimulated M0 macrophages.**

**Figure S3. Impact of LPS stimulation on the transcription of *GPR813, IL1B, TNFA*, and *IL6* in THP-1 monocytes.**

**Figure S4. Effects of lauroyl tryptamine in LPS-stimulated M0 macrophages.** **Genes in the NFKB pathway: *NFKIA* and *RELA.***

**Table S1. List of genes and their corresponding primers.**

| **Gene name** |  | **Sequence (5’ - 3’)** | **Synonym** |
| --- | --- | --- | --- |
| ***GAPDH*** | Forward | AAGGTGAAGGTCGGAGTCAA |  |
|  | Reverse | AATGAAGGGGTCATTGATGG |  |
| ***ACTB*** | Forward | AGAGCTACGAGCTGCCTGAC |  |
|  | Reverse | AGCACTGTGTTGGCGTACAG |  |
| ***IL6*** | Forward | AGTGAGGAACAAGCCAGAGC |  |
|  | Reverse | GTCAGGGGTGGTTATTGCAT |  |
| ***IL8*** | Forward | TCCTGATTTCTGCAGCTCTGT | ***CXCL8*** |
|  | Reverse | AAATTTGGGGTGGAAAGGTT |  |
| ***TNFΑ*** | Forward | AGATGATCTGACTGCCTGGG |  |
|  | Reverse | CTGCTGCACTTTGGAGTGAT |  |
| ***IL10*** | Forward | GCTGTCATCCATTTCTTCCC |  |
|  | Reverse | CTCATGGCTTTGTAGATGCCT |  |
| ***IL1Β*** | Forward | AAGCCCTTGCTGTAGTGGTG |  |
|  | Reverse | GAAGCTGATGGCCCTAAACA |  |
| ***NFKBIA*** | Forward | AAAGCCAGGTCTCCCTTCAC |  |
|  | Reverse | CAGCAGCTCACCGAGGAC |  |
| ***RELA*** | Forward | GGTCCGCTGAAAGGACTCTT |  |
|  | Reverse | GAATTCCAGTACCTGCCAGA |  |
| ***IL2*** | Forward | ACCTCAACTCCTGCCACAAT |  |
|  | Reverse | GCCTTCTTGGGCATGTAAAA |  |
| ***GPR183*** | Forward | ACCACCGCTTTGCCTACACGAA | *EBI2* |
|  | Reverse | CACCACAGCAATGAAGCGGTCA |  |
